# Steroid implants for the induction of vitellogenesis in feminized European silver eels (*Anguilla anguilla* L.)

**DOI:** 10.3389/fgene.2022.969202

**Published:** 2022-08-17

**Authors:** Arjan P. Palstra, Lotte J. Bouwman, Pauline Jéhannet, Leo Kruijt, Henk Schipper, Marco H. Blokland, William Swinkels, Leon T. N. Heinsbroek, P. Mark Lokman

**Affiliations:** ^1^ Animal Breeding and Genomics, Wageningen University and Research, Wageningen, Netherlands; ^2^ Experimental Zoology Group, Wageningen University and Research, Wageningen, Netherlands; ^3^ Wageningen Food Safety Research, Wageningen University and Research, Wageningen, Netherlands; ^4^ DUPAN Foundation, Wageningen, Netherlands; ^5^ Wageningen Eel Reproduction Experts B.V, Wageningen, Netherlands; ^6^ Department of Zoology, University of Otago, Dunedin, New Zealand

**Keywords:** aquaculture, eel reproduction, 11-ketotestosterone (11KT), FSH receptor, steroidogenesis, liquid chromatography mass spectrometry (LCMS)

## Abstract

Assisted propagation of the European eel will lead to a closed production cycle supplying the aquaculture industry with juvenile glass eels. Females require long-term weekly treatment with pituitary extract (PE), which is stressful and causes abnormalities in oogenesis. We tested the effects of 17α-methyltestosterone (17 MT), as potent androgen activating the androgen receptor, and 17β-estradiol (E2), as an inducer of vitellogenesis, to shorten the duration of PE treatment.Four groups of feminized eels were subjected to a simulated migration and subsequent injection with implants containing 17 MT (17 MT-group), E2 (E2-group) or 17 MT plus E2 (17 MT + E2-group) to test for synergistic effects, or without any steroids as controls (C-group). The effects of a 2-months treatment were investigated by determining the eye index (EI), hepatosomatic and gonadosomatic index (HSI and GSI, respectively), plasma steroid concentrations by liquid chromatography mass spectrometry (LCMS), gonadal histology, expression of androgen receptors a and b (*ara*, *arb*); estrogen receptor 1 (*esr1*); FSH receptor (*fshr*); vitellogenin receptor (*vtgr*) and aromatase (*cyp19*), and the required number of weekly PE injections to fully mature. For many parameters, both the 17 MT and E2 groups showed an increase vs. controls, with the 17 MT + E2 group showing a synergistic effect, as seen for EI, GSI (3.4 for 17 MT and for E2, 6.6 for 17 MT + E2), oocyte diameter and *ara*, *arb* and *esr1* expression. Concentrations of almost all focal steroids decreased with simulated migration and steroid treatment. Only eels of the 17 MT-group showed increased expression of *cyp19* and of *fshr*, while *fshr* expression increased 44-fold in the 17 MT + E2 group, highlighting that co-implantation is most effective in raising *fshr* mRNA levels. Specific for eels of the E2 groups were vitellogenesis-associated changes such as an increase of HSI, plasma E2, and presence of yolk in the oocytes. Steroid treatments reduced the duration of PE treatment, again synergistically for co-implantation. In conclusion, E2 is necessary to start vitellogenesis, but 17 MT has specific effects on *cyp19* and *fshr* expression. The combination is necessary for synergistic effects and as such, steroid implants could be applied in assisted reproduction protocols for European eel to improve oocyte quality leading to the production of more vital larvae.

## 1 Introduction

The silvering of eels precedes the initiation of puberty and activation of the brain-pituitary-gonad (BPG) axis (Weltzien et al., 2009). 11-Ketotestosterone is the major silvering-initiating androgen (11KT; Rohr et al., 2001; Thomson-Laing et al., 2018) and it plays a key role in lipid accumulation in previtellogenic oocytes of shortfinned eels, *A. australis* (Damsteegt et al., 2015), and Japanese eels, *A. japonica* (Endo et al., 2011). In captive European eel, dopaminergic inhibition of the pituitary restricts the synthesis and release of follicle-stimulating hormone (Fsh), essential for the induction of vitellogenesis (Vidal et al., 2004). For vitellogenesis to commence, Fsh needs to bind to its ovarian receptor (Fshr) to increase ovarian aromatase (Cyp19) activity, which in turn converts testosterone into 17β-estradiol (E2) (Nagaharna et al., 1995; Montserrat et al., 2004). When diffused into the circulation of the eel, E2 binds to its hepatic nuclear estrogen receptor (Esr1) and induces the synthesis of the phospholipoglycoprotein vitellogenin (Vtg) (Wallace, 1985). Vitellogenins bind to their receptor (Vtgr) on the oocyte surface, after which Vtg is cleaved into yolk proteins (Wallace, 1985) that later serve as a nutrient source for the developing embryos and larvae (Sire et al., 1994; Lubzens et al., 2010).

Despite intensive research efforts (reviewed by Palstra and van den Thillart, 2009; Mordenti et al., 2019; Tomkiewicz et al., 2019; Asturiano et al., 2020), the life cycle of European eel in captivity is still not closed. In our facilities, we simulate life cycle events to cover the trajectory from wild juvenile glass eel up to larval production. Broodstock conditioning is achieved by the feminization of young elvers (Chai et al., 2010), later followed by a simulation of the oceanic reproductive migration (Palstra and van den Thillart, 2010). Simulated migration stimulates the silvering of farmed eels (Mes et al., 2016) and feminized eels (Böhm et al., 2016). After migration, the female eels receive weekly carp pituitary extract (CPE) injections to induce vitellogenesis (Kagawa et al., 1997; Ohta et al., 1997). Oocyte maturation and ovulation are induced by injection of 17,20β-dihydroxy-4-pregnen-3-one (DHP) (Kagawa et al., 1995; Palstra et al., 2005; Tanaka, 2014). In our facilities, we produce, from three different females, up to 100,000 larvae each week that stay alive for up to 25 days post hatch. Still, egg and larval quality is often compromised, reflected in deformities and high mortality during the first week after hatching, thus preventing the execution of large-scale feeding trials. Low egg and larval quality is a persisting reproductive bottleneck for European eel (Politis et al., 2017, 2021; Kottmann et al., 2020a; Kottmann et al., 2020b), and indeed, even still for Japanese eels (Okamura et al., 2020) for which the life cycle has been closed and a fifth generation has been produced (Tsukamoto, 2019). There is an urgent need for improved techniques and protocols to obtain high quality European eel eggs and resilient larvae, and ultimately, to close the cycle of European eel in captivity. The European eel population has declined from 1980 to 2011 and, although this decline has stopped for over a decade now, there are no clear signs of Europe-wide recovery yet (ICES, 2021). A closed production cycle would supply the aquaculture industry with juvenile glass eels and could release fishing pressure on the wild population.17α-Methyltestosterone (17 MT) is an androgen that activates the eel androgen receptor with similar potency as 11 KT (*A. japonica*; Todo et al., 1999). Steroid implants containing 11 KT or 17 MT successfully stimulated lipid accumulation and increased oocyte size in silver shortfinned eels that had already initiated vitellogenesis (Lokman et al., 2015; Thomson-Laing et al., 2019). Treatment with 17 MT-containing cholesterol-cellulose implants prior to starting CPE injections increased fecundity, oocyte quality, hatching rate and survival of larvae in European eel (Di Biase et al., 2017; Mordenti et al., 2018). Thomson-Laing et al. (2019) demonstrated that incorporation of both 11 KT and E2 in one implant elicited a synergistic effect in shortfinned eel, such that only co-treatment, but not separate 11 KT or E2 treatment, induced Vtg uptake and yolk deposition.

In the present study, we investigated the effects of 17 MT, as potent androgen activating the androgen receptor, and 17β-estradiol (E2), as inducer of vitellogenesis, on the sexual maturation in feminized European eels. We used an experimental set-up in which feminized eels were first subjected to simulated migration and then assigned to one of four groups that were injected with implants containing 17 MT or E2 (experimental groups), with17 MT + E2 (positive controls), or implants without any steroids (negative controls). Effects of a 2-months steroid treatment were investigated by assessment of the eye index and gonadosomatic index, indicating the maturity stage, plasma steroid concentrations as determined by liquid chromatography mass spectrometry (LCMS), gonadal histology, and RT-PCR of the genes encoding androgen receptors a and b (*ara* and *arb*), estrogen receptor 1 (*er1*), Fsh receptor (*fshr*), vitellogenin receptor (*vtgr*) and aromatase (*cyp19*). Eels of the different treatments were matured by weekly CPE injections to determine the percentage of eels that reached full maturity and the weeks that were required to reach it.

## 2 Materials and methods

### 2.1 Ethics

Experiments were approved by the Dutch Central Committee for Animal Experimentation (CCD nr. AVD401002017817) and the Animal Experimental Committee of Wageningen University (IvD nr. 2017.D.0007.003).

### 2.2 Experimental eels, feminization and simulated migration

**FIGURE 1:**
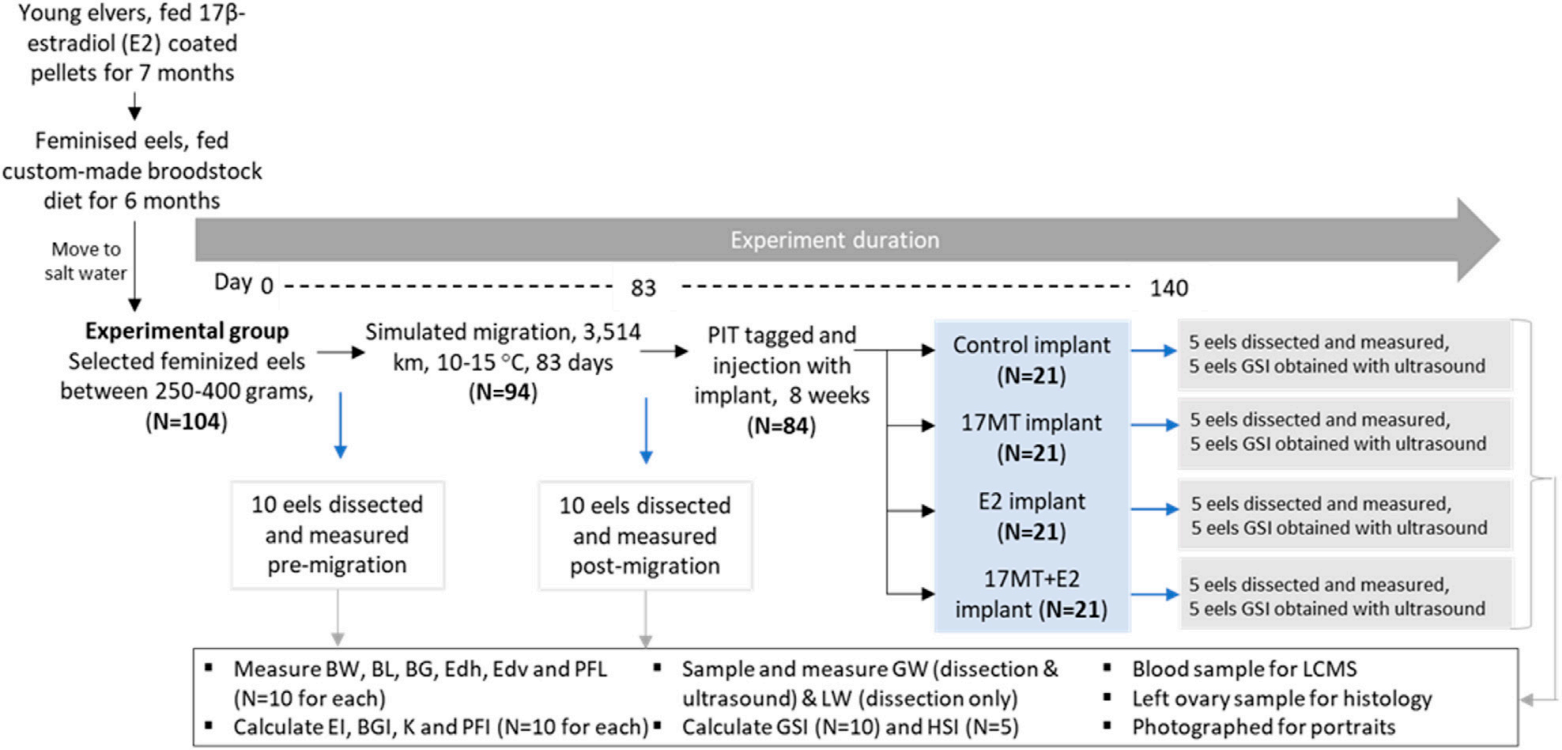
Experimental set-up. The 16 remaining eels per implant treatment group were matured by weekly injections with carp pituitary extract. Abbreviations: BW = body weight; BL = body length; BG = body girth; GW = gonad weight; LW = liver weight; EDh = Eye diameter horizontal; EDv = eye diameter vertical; PFL = pectoral fin length; K= Fulton’s condition factor; BGI = body girth index; GSI = gonadosomatic index; HSI = hepatosomatic index; EI = eye index; PFI = pectoral fin index; 17 MT = 17α-methyltestosterone; E2 = 17β-estradiol; LCMS = liquid chromatography mass spectrometry.

Juvenile glass eels were reared for 1 month up to young elvers at eel farm Palingkwekerij Koolen (Bergeijk, Netherlands) and then transferred to the Wageningen University and Research animal research facilities (CARUS, Wageningen, Netherlands). First, the elvers were feminised by feeding them with E2-coated pellets over a 7 month-period (Chai et al., 2010; Ijiri et al., 2011; Figure 1). After an additional 6 months of feeding with a custom-made broodstock diet, eels of 250–400 g (*N* = 104) were selected (Figure 1), transferred to seawater and no longer fed. Eels were acclimated to these conditions for 7 weeks. A group of *N* = 10 eels was anaesthetized (2-phenoxyethanol, 0.2 ml L^−1^), measured and dissected (pre-migration group) as described in Section 2.4. The remaining 94 eels were anaesthetised, photographed and subjected to simulated migration: constant swimming in seawater, in the dark and at daily alternating temperatures between 10 and 15°C (based on Mes et al., 2016; Figure 1). After 83 days of migration and covering 3,500 km, a second group of *N* = 10 eels was anaesthetized, measured and dissected (post-migration group). Remaining eels (*N* = 84) were anaesthetized, photographed, PIT-tagged (Passive Integrated Transponder, Trovan, Aalten, Netherlands; size 2 × 12 mm; intramuscular injection close to the dorsal fin) and injected with implants (Figure 1).

### 2.3 Steroid implant treatment

Experimental eels (*N* = 84) were injected intraperitoneally with a cholesterol-cellulose implant. Implants contained 5 mg 17 MT per fish (17 MT-group, *N* = 21 eels), 2 mg E2 per fish (E2-group, *N* = 21 eels), both 5 mg of 17MT and 2 mg E2 per fish (17 MT + E2-group, *N* = 21 eels), or no steroid (controls, C-group, *N*= 21 eels). After 2 months of treatment, *N* = 5 similar-sized fish per treatment group were anaesthetized, photographed, measured, scanned by ultrasound and dissected as described in Section 2.6 (Figure 1), while *N* = 5 others were also anaesthetized, photographed, measured and scanned, but not dissected.

### 2.4 Treatment with carp pituitary extract

Remaining eels (*N* = 16 per implant treatment group) were used to induce full sexual maturation (oocyte hydration response indicated by body weight increase of >10%) by weekly injections with carp pituitary extract (CPE; 20 mg kg^−1^; Palstra et al., 2005). The percentage of eels per group that reached this stage was determined as well as the number of weekly CPE injections that were required.

### 2.5 Biometry

Eel heads were photographed and images were aligned in Photoshop (Adobe Systems, Mountain View, United States ) to illustrate differences in external appearance between treatment groups. Eels were measured for body length (BL), body weight (BW), body girth (BG), horizontal eye diameter (EDh), vertical eye diameter (EDv), and pectoral fin length (PFL). From these parameters, the Fulton’s condition factor (K), body girth index (BGI), eye index (EI; Pankhurst, 1982), and pectoral fin index (PFI) were calculated (Supplementary Material S1).

### 2.6 Dissection and ultrasound

Eels were dissected to determine gonad weight (GW) and liver weight (LW), and to calculate the gonadosomatic index (GSI) and hepatosomatic index (HSI), respectively (formulas in Supplementary Material S1).

A fragment of ovarian tissue was sampled from a standardised location of each eel and split into two: one part was preserved in RNA later stabilization solution (Ambion) at −20°C for subsequent RT-PCR, and one part was fixed in formalin overnight and then stored in 70% ethanol for 24 h before histological analysis.

From these eels, and from an additional *N* = 5 eels per treatment group, GSI was determined non-invasively by ultrasonography using a compact and portable system (MyLabFive™Vet with a LA435 probe, Esaote, Genoa, Italy; Jehannet et al., 2017). GSI was calculated following Bureau du Colombier et al. (2015):

$$\mathrm{GSI}=(\log\left(GM_{E\left(LM\right)}\right)=0.07062+0.55102\times \log\left(GL_{E}\right)+1.11327\times \log\left(\mathrm{mean gonad area}\right)+\varepsilon$$

GL:gonad length,GM:gonad mass,ε as a variance σ2=0.05

The calculated GSI values were compared with the GSI values of the dissected eels to assess the accuracy of the calculations.

### 2.7 Blood plasma concentrations of steroids by liquid chromatography mass spectrometry

Blood samples were taken from the caudal artery with a heparin-flushed syringe and immediately put on ice. Plasma was separated by centrifugation (10,000 rpm, 5 min, 4°C) and stored at −80°C. A slightly modified UHPLC-MS/MS method as described by Blokland et al. (2017) was used to detect endogenous free steroids in serum. In short, 900 μl water was added to 100 μl serum. The mixture was centrifuged and subjected to solid-phase extraction using an Oasis HLB 96-well SPE plate (Waters, Milford, CA, United States ). After elution, derivatization of hydroxy groups attached to the A-ring was performed with picolinic-acid. Chromatographic separation using water (containing 0.1% formic acid) acetonitrile gradient was performed on a Waters BEH C18 column (1.7 µm particles, L = 10 cm, id 2.1 mm). The mass-spectrometric analysis was performed on a Xevo TQS mass spectrometer (Waters) in positive and negative electrospray mode. The lower detection limit was 0.1 ng ml^−1^.

### 2.8 Histological analysis

Gonadal samples were embedded in paraffin wax and cut into 5 µm sections using a motorized rotary microtome (HM 350, Microtom). Per sample, two slides containing 6 sections, each at least 30 µm apart, were stained for nuclei and cytoplasm using Mayer’s haematoxylin—eosin staining method. Sections were imaged using a Leica DM6b upright microscope (Wageningen, Netherlands). For each sample, the 10 largest oocytes with a visible central nucleus were selected. Oocytes were measured for their diameter and the diameter of the 10 largest lipid droplets in each of these oocytes was measured using the image-processing software ImageJ (Schneider et al., 2012).

### 2.9 RNA isolation, quality assessment and cDNA synthesis

Total RNA was isolated from RNA*later*-preserved tissue portions of 0.01 g (Cf., L182) and subsequently purified (Qiagen, RNeasy Plus Universal Mini Kit) according to manufacturers’ instructions. RNA quantity (>500 ng ml^−1^) and purity (>2.0 260/280 ratio) were determined by Nanodrop. Supplementary RNA quality assessment (RIN-values > 7.5 were considered as acceptable) was done using the 2,100 Bioanalyzer 6,000 Nano Assay Kit (Agilent Technologies, Santa Clara, CA, United States ). When calculation of RIN values was impossible due to high 5 S rRNA signals, the 28S/18 S ratio was considered as quality indicator for those samples (values > 1.9 were considered as acceptable). Total RNA was reverse transcribed (Applied Biosystems™ High-Capacity cDNA Reverse Transcription Kit) according to manufacturers’ instructions and resulting cDNA was diluted to a concentration of 1/50 ng cDNA µl^−1^ in MilliQ (250 ng RNA µl^−1^ solution was used to transcribe into 4 µl cDNA, or RNA-equivalent, that was diluted in 196 µl MilliQ).

### 2.10 Comparative RT-PCR analysis

Target genes were selected and primer sequences were used as published by Jéhannet et al. (2019) for *Anguilla*, or as designed for *A. australis* by Setiawan and Lokman (2010), Setiawan et al. (2012) and Zadmajid et al. (2015); Table 1. Messenger RNA levels were quantified employing a Quantstudio 5 Real-Time PCR System (ThermoFisher Scientific). Real-time polymerase chain reactions were performed for a 20 µl mixture containing cDNA (5 μl, 1/50 ng μl^−1^), primers (1 μl, 5 µM each), SensiFAST SYBR Lo-ROX Kit (10 μl; Bioline, Luckenwalde, Germany) and MilliQ (4 µl). Samples were run as singletons twice in 96-well plates, containing primers for the two target genes, the *elf1* reference gene and two negative controls for each gene. The “pre-migration” group was set as calibrator. RT-PCR assays were run using a temperature profile starting at a hold stage (95°C, 2 min), followed by 30–40 cycles of denaturation (95°C, 15 s), and annealing/extension (60°C, 30 s and 72°C, 5 s) and ending with the melt curve stage (95°C for 1 s, followed by 60°C for 20 s) gradually heating (0.1°C s^−1^) to 95°C. Resulting melting curves were analysed to account for reaction specificity and absence of primer-dimers artefacts. Primer efficiencies were determined using a dilution series to generate standard curves for the housekeeping gene and for each target gene.

**TABLE 1:** Primers for each of the targeted genes, where Abv: gene abbreviation; Accession number: the Genbank accession number for *A. anguilla*; G: sequence obtained from the *A. anguilla* genome by Jéhannet et al. (2019); Amplicon size: the PCR product size in base pairs (bp) of nucleotides; annealing temp (°C): annealing temperature of target gene; Reference: the literature source from which the original primers were obtained from.

Abv.	Target gene	Accession number	Primers (5′−3′)	Amplicon size (bp)	Annealing temp (°C)	References
*ara*	Androgen receptor a	FR668031	FW: AGG​AAG​AAC​TGC​CCC​TCT​TG	90	62	Setiawan et al. (2012)
			RV: ATT​TGC​CCG​ATC​TTC​TTC​AG			
*arb*	Androgen receptor b	FR668032	FW: GCT​TGG​AGC​TCG​AAA​ATT​GA	98	62	Setiawan et al. (2012)
			RV: TTG​GAG​AGA​TGC​ACT​GGA​TG			
*cyp19*	Aromatase Cytochrome P450	KF990052	FW: CGC​ACC​TAC​TTT​GCT​AAA​GCT​C	137	62	Jéhannet et al. (2019)
			RV: AGG​TTG​AGG​ATG​TCC​ACC​TG			
*elf1*	Elongation factor-1	EU407825	FW: CCC​CTG​CAG​GAT​GTC​TAC​AA	152	64	Setiawan and Lokman, (2010)
			RV: AGG​GAC​TCA​TGG​TGC​ATT​TC			
*esr1*	Estrogen receptor 1	LN879034	FW: GGCATGGCCGAGATTTTC	116	62	Jéhannet et al. (2019)
			RV: GCA​CCG​GAG​TTG​AGC​AGT​AT			
*fshr*	Follicle stimulating hormone receptor	LN831181	FW: CCT​GGT​CGA​GAT​AAC​AAT​CAC​C	173	63	Zadmajid et al. (2015)
			RV: CCT​GAA​GGT​CAA​ACA​GAA​AGT​CC			
*vtgr*	Vitellogenin receptor	G	FW: TCTGAACGAACCCCAGGA	140	59	Jéhannet et al. (2019)
			RV: TTT​GGG​GAG​TGC​TTG​TTG​A			

### 2.11 Data analyses

K, BGI, EI, PFI, GSI, and HSI data were analyzed by comparing means between groups using the non-parametric Kruskal-Wallis test to account for varying sample sizes across groups and complications inherent to proportion-based data, with Benjamini-Hochberg correction for false discovery rate. The same test was used for differences in blood plasma concentrations of steroids, gene expression levels as determined by fold change (2–∆∆Ct) between groups (Livak and Schmittgen, 2001) and numbers of weeks that were required to induce maturation and of matured eels for each group. Pairwise Mann Whitney U-tests were employed to estimate the significant differences between treatment groups. Oocyte diameter and lipid droplet diameter data obtained from the histological images were normally distributed and tested for significant differences by ANOVA with Tukey post-hoc correction. Their correlation was analyzed with a two-tailed Pearson correlation test. *p*-values < 0.05 were considered to indicate statistically significant differences between groups. Data analyses were done using R-studio (version 4.0.0) and SPSS 25.0.

## 3 Results

### 3.1 Portrait images

**FIGURE 2:**
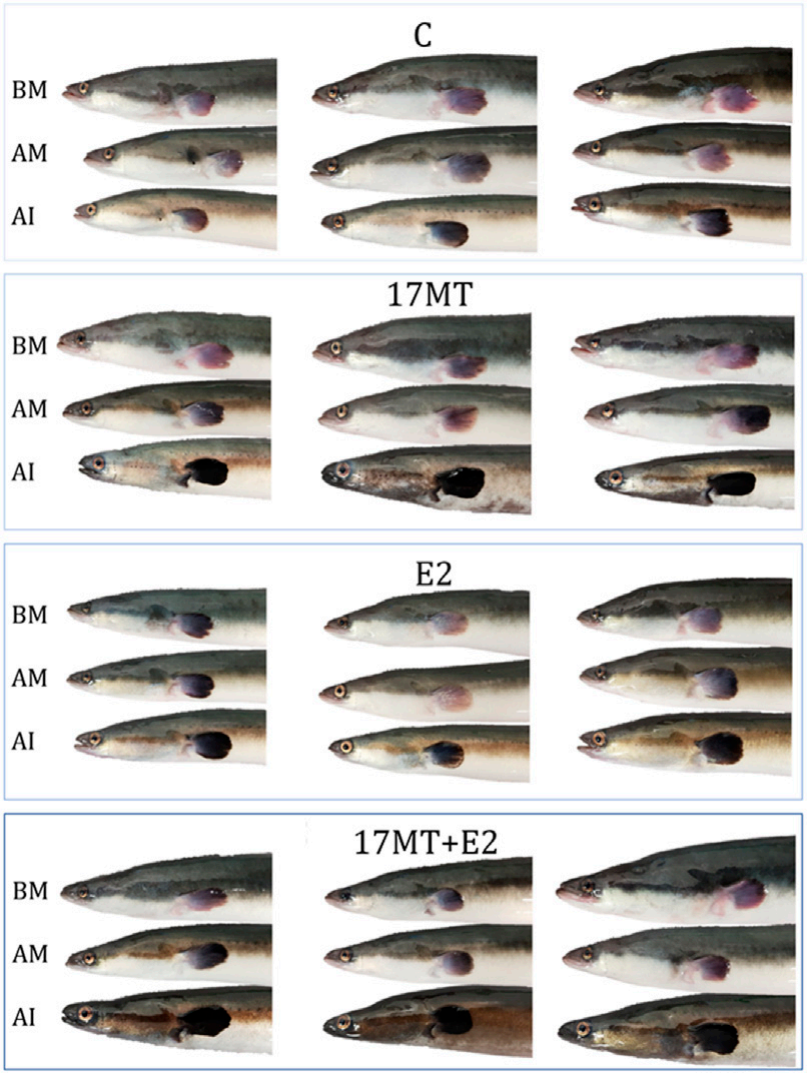
Examples of portraits of the same eels (*N* = 3 representative eels per group) before migration (BM), after migration (AM) and after 8-weeks implant-treatment (AI). Eels were subjected to simulated migration to induce silvering: constant swimming in the dark at daily alternating temperatures between 10 and 15°C, for 83 days and covering 3,500 km. After migration, eels were equally divided over four implant treatment groups. The controls received a sham implant; the E2 group an implant with 17β-estradiol; the 17 MT group an implant with 17α-methyltestosterone; and the 17 MT + E2 group received an implant with 17 MT and E2. Changes in external appearance such as eye enlargement and pectoral fin darkening were observed after migration and more advanced after implant treatment.

The images of individual eels at the different stages of the experiment (Figure 2) clearly showed that eels underwent extensive external changes. Firstly, eye size increased after simulated migration, and then increased further during the implantation period but independently of the kind of implant. Secondly, darkening of the pectoral fin and dorsal side of the body occurred. Darkening of the pectoral fin was initiated during simulated migration but progressed after implant treatment. Darkening of the skin was observed first on the dorsal side of the eels after migration. After implant treatment, eels of the 17 and 17 MT + E2 groups showed darker skin on the dorsal side than the C and E2 groups, and also a bronze colouring of the ventral side of the body in what appeared as the most advanced maturation stages. Thirdly, the head shape changed from acute (pointy) to spheric after migration and particularly after the implantation period across all experimental groups.

### 3.2 Biometry

Significant differences across all groups were observed for K (*p* < 0.001) and BGI (*p* = 0.01) that decreased, and EI (*p* < 0.0001) and PFI (*p* = 0.01) that increased (Table 2). A significant effect of simulated migration was associated with decreased K and increased EI (Table 2). A significantly increased EI was apparent for the four implant treatment groups (Table 2).

**TABLE 2:** Biometry (Mean ± SEM). Measurements on eels dissected before (pre) simulated migration (*N* = 10) and after (post) simulated migration (*N* = 10), and eels after implant treatment (*N* = 10) for each treatment group with C = control with sham implant; 17 MT = 17α methyltestosterone implant; E2 = 17β-estradiol implant, and 17 MT + E2 = the co-implantation. Abbreviations: BL = body length; BW = body weight; K= Fulton’s condition factor; BGI = body girth index; EI = eye index; PFI = pectoral fin index. K, BGI, EI and PFI were tested for statistical differences between groups which are indicated by letters a-d.

	BL	BW	K	BGI	EI	PFI
simulated migration						
Pre-migration	57 ± 1	363 ± 21	0.20 ± 0.00^a^	0.19 ± 0.00^a^	6.61 ± 0.22^a^	0.37 ± 0.02^ac^
Post-migration	57 ± 1	330 ± 10	0.18 ± 0.00^b^	0.18 ± 0.00^b^	7.70 ± 0.17^b^	0.36 ± 0.02^a^
implant treatment						
C	56 ± 1	304 ± 12	0.17 ± 0.00^b^	0.18 ± 0.00^ab^	9.81 ± 0.33^c^	0.35 ± 0.01^a^
17 MT	57 ± 1	316 ± 9	0.17 ± 0.01^b^	0.19 ± 0.00^a^	10.81 ± 0.26^days^	0.41 ± 0.02^b^
E2	56 ± 1	297 ± 13	0.17 ± 0.00^b^	0.19 ± 0.00^ab^	10.27 ± 0.35^cd^	0.41 ± 0.02^bc^
17 MT + E2	57 ± 1	323 ± 7	0.17 ± 0.01^b^	0.20 ± 0.00^a^	11.36 ± 0.44^days^	0.39 ± 0.02^abc^

### 3.3 Dissection and ultrasound

**FIGURE 3:**
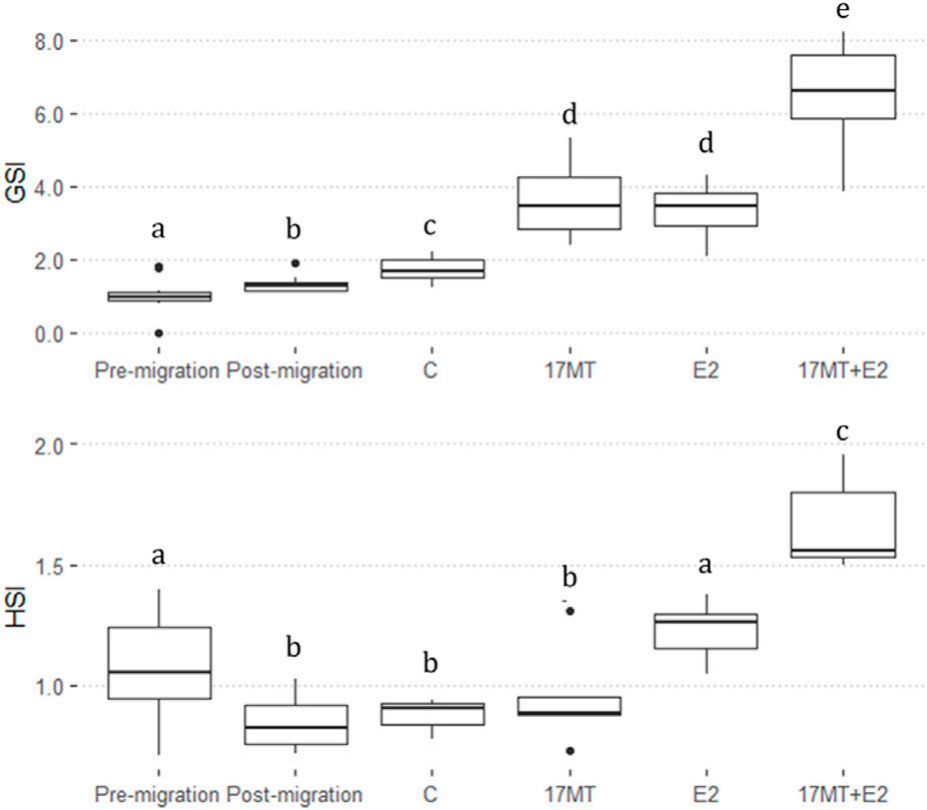
Gonadosomatic index (GSI) and Hepatosomatic index (HSI) before-and after simulated migration, and after 2 months of steroid-implant treatment, with: control (C group; GSI, N = 10; HSI, *N* = 5); 17α-methyltestosterone (17 MT group; GSI, *N* = 10; HSI, *N* = 5); 17β-estradiol (E2 group; GSI, *N* = 10; HSI, *N* = 5), and co-implantation of 17 MT and E2 (17 MT + E group; GSI, *N* = 10; HSI, *N* = 5). At each stage, N = 5 eels were dissected for body weight (BW), gonad weight (GW) and liver weight (LW). Another *N* = 5 eels were scanned to calculate GW using ultrasound.

GSI was significantly higher in the steroid implant-treated eels (17 MT: 3.47 ± 0.28 (*N* = 5); E2: 3.45 ± 0.47 (*N* = 5); 17 MT + E2: 6.59 ± 0.46 (*N* = 5)) than in the pre-migration-, post-migration-, or implant control groups (Pre-migration: 1.13 ± 0.13 (*N* = 10); Post-migration: 1.32 ± 0.08 (*N* = 10); C: 1.88 ± 0.11 (*N* = 5) (*p* < 0.0001). GSI calculations applying ultrasound on the same eels just before dissection [C: 1.63 ± 0.15 (*N* = 5); 17 MT: 3.19 ± 0.87 (*N* = 5); E2: 3.69 ± 0.41 (*N* = 5); 17 MT + E2: 6.48 ± 1.29 (*N* = 5)] closely matched these GSI values. Therefore, the ultrasound and dissection data were pooled providing *N* = 10 for GSI determination for each of the treatment groups. The pooled GSI values of the control group were significantly greater than of the post-migration group. A significant overall treatment effect (*p* < 0.001; Figure 3) of the implants was apparent.

Hepatosomatic index (HSI) was significantly lower after migration. Significant differences were observed between all treatment groups (Pre-migration: 1.07 ± 0.07 (*N* = 10); Post-migration: 1.32 ± 0.08 (*N* = 10); C: 0.88 ± 0.03 (*N* = 5); 17 MT: 1.23 ± 0.06 (*N* = 5); E2: 0.95 ± 0.09 (*N* = 5); 17 MT + E2: 1.67 ± 0.09 (*N* = 5); *p* < 0.0001) (Figure 3).

In general, eels of the 17 MT + E2 group had the highest values of GSI and HSI (Figure 3).

### 3.4 Quantification of plasma steroid concentrations by liquid chromatography mass spectrometry

**FIGURE 4:**
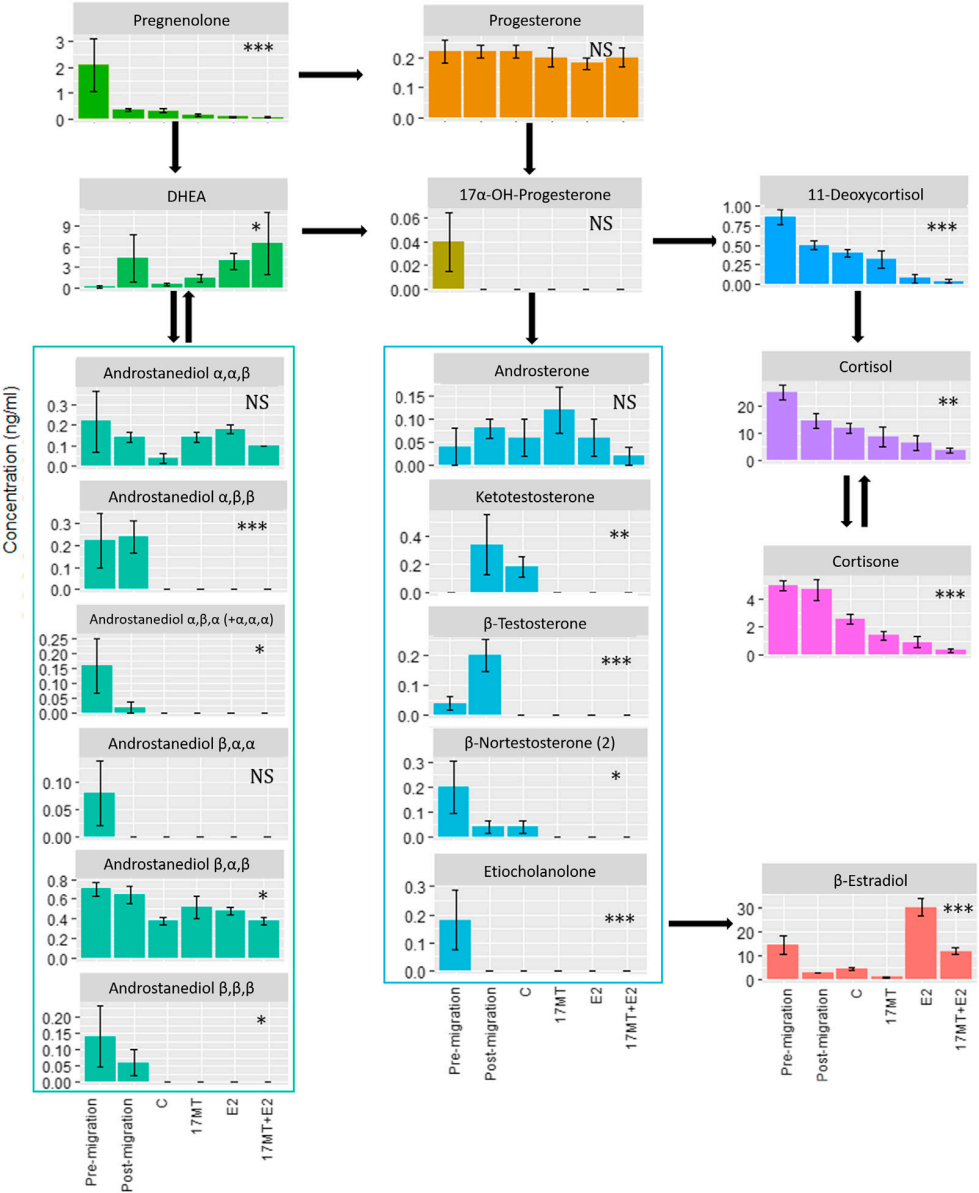
Blood plasma concentrations of steroids as determined by LCMS. Twenty out of thirty-three steroids showed detectable levels of which fifteen showed significant differences between treatment groups: pre-(*N* = 5) and post-simulated migration (*N* = 5); control (C group; *N* = 5); 17α-methyltestosterone (17 MT group; *N* = 5); 17β-estradiol (E2 group; *N* = 5), and co-implanted 17 MT and E2 (17 MT + E2 group; *N* = 5). Significant differences are indicated with *p < 0.05, **p < 0.01 and ***p < 0.001. NS means non-significant.

Among 33 focal steroid hormones, thirteen did not show detectable levels: 17α-OH-pregnenolone; 11-dehydrocorticosterone; 11-deoxycorticosterone; 4-androstene-3,17-dione; α-testosterone; 5α-androstane-17β-ol-3-one (5α-dihydrotestosterone—5α-DHT); 5β-androstane-17α-ol-3-one; 5α-androstanedione; 5β-androstane-3ß,17α-diol (androstanediol β,β,α); nor-epiandrosterone; 19-norandrostenedione; β-nortestosterone and estriol. Twenty steroids circulated at detectable levels, fifteen of which showed significant differences between the treatment groups (Figure 4). There was a strong overall trend for E2 and/or 17 MT to reduce the concentrations of steroids in blood—indeed, across the 20 measurable steroids, and excluding E2, dehydroepiandrosterone (DHEA) was the only steroid metabolite to show an increase in steroid-implanted eels when compared to blank controls; the majority of candidate steroids, representing C21, C19, and C18 steroid classes, were below or near the limit of detection (Figure 4).

Interestingly, notable changes in steroid profiles were evident when comparing eels prior to, and after the simulated migration. Thus, levels of pregnenolone, the first steroid in the steroid biosynthetic cascade, were high (but variable: 2.1 ± 2.3 ng ml^−1^) prior to, and decreased to 0.4 ± 0.1 ng ml^−1^) on completion of, simulated migration. Levels of E2 (14.4 ± 9.0 ng ml^−1^) and cortisol (24.8 ± 6.1 ng ml^−1^), similarly, were higher before than after swimming (2.92 ± 0.2 and 14.5 ± 5.7 ng ml^−1^, respectively), while those of remaining steroids, including testosterone and 11-ketotestosterone, were low, close to the detection limit. The androgen 5β-androstane-3α,17β-diol deserves a mention as its levels (∼ 0.7 ng ml^−1^) were the highest among the detected androgens and indeed, 5β-reduced focal androstanes seemed more abundant than their 5α-reduced analogues. After simulated migration, levels of testosterone, 11-ketotestosterone and, particularly, DHEA (∼4 ng/ml^−1^) increased. In contrast, the plasma concentration of 5β-androstane-3α,17β-diol remained unchanged, while levels of E2 and cortisol notably decreased (Figure 4). Levels of all focal steroids decreased after subsequent sham pellet-implantation and maintenance for 2 months (Figure 4).

### 3.5 Histology

**FIGURE 5:**
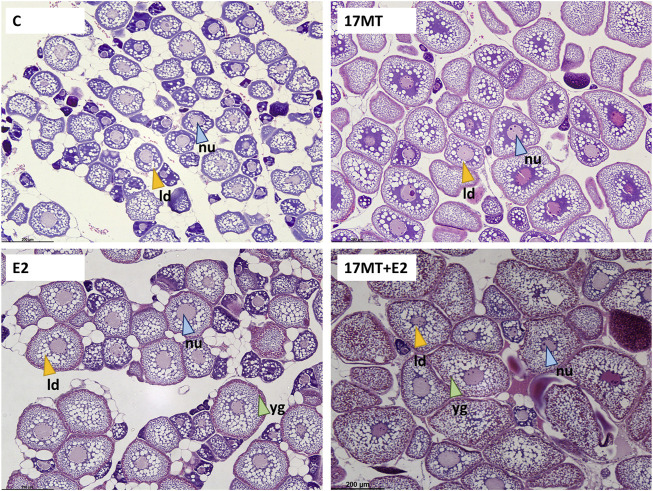
Oocytes in the eel ovaries after receiving a 2-months steroid-implant treatment, with: control (C group; *N* = 10); 17α-methyltestosterone (17 MT group; *N* = 10); 17β-estradiol (E2 group; *N* = 10), and co-implanted 17 MT and E2 (17 MT + E2 group; *N* = 10). Scale = 200 µm. Stain: Mayer’s haematoxylin and eosin. The white globules indicate the (empty) lipid droplets (ld—yellow arrows), bright pink globules are yolk globules (yg—green arrows). A central nucleus (nu—blue arrows) with perinucleoli can be observed. For ovaries of eels of all groups, small developing oocytes in the perinucleolus stage and early lipid droplet stage could still be observed.

**FIGURE 6:**
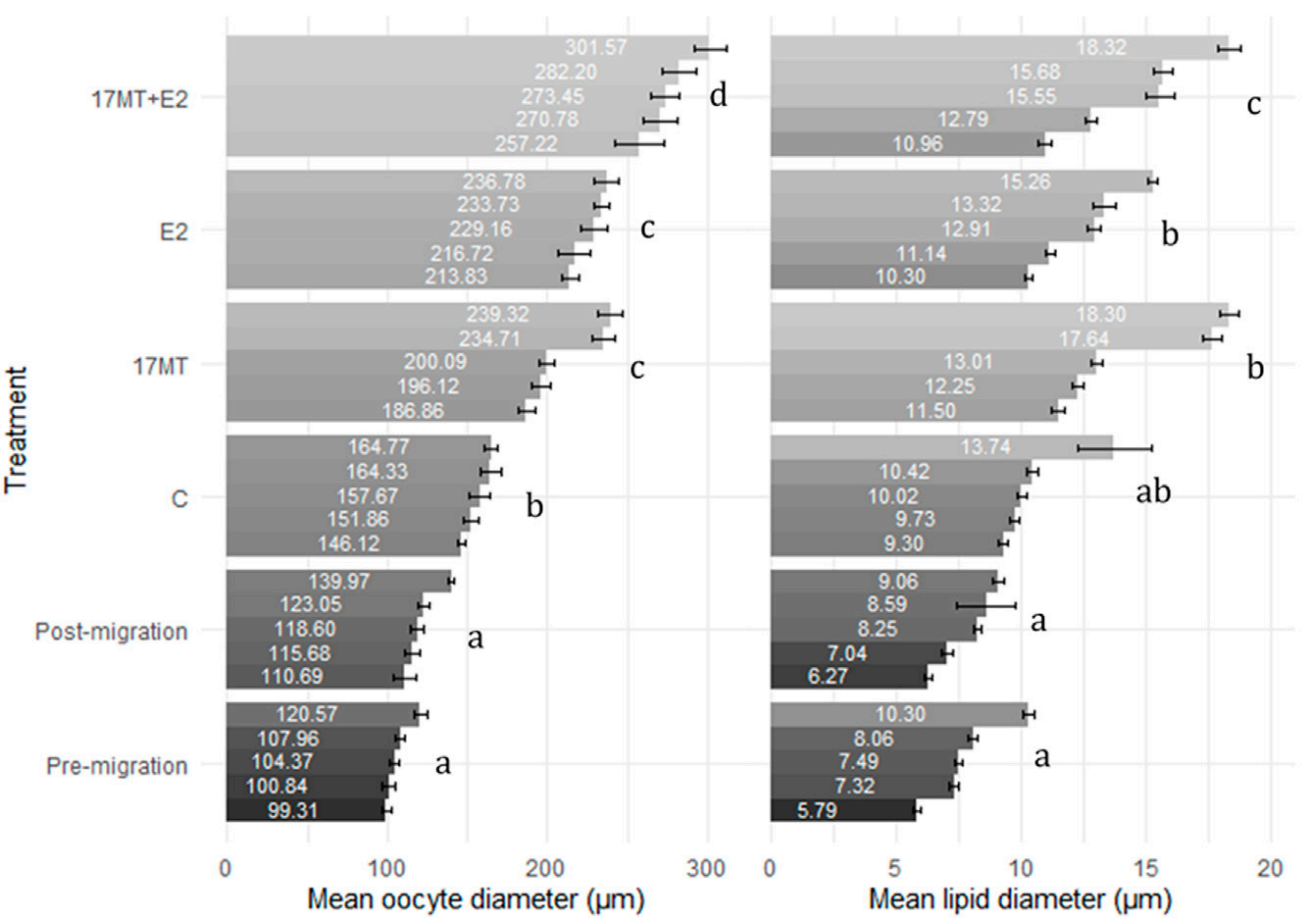
Oocyte (*N* = 10 oocytes per eel) and lipid droplet diameter (*N* = 10 lipid droplets per oocyte) for ovarian tissue obtained by analyzing the histological images. Mean diameter is presented for each eel with standard error bar. Six treatment groups are presented: pre-(*N* = 5 eels) and post-simulated migration (*N* = 5 eels); control (C group; *N* = 5 eels); 17α-methyltestosterone (17 MT group; *N* = 5 eels); 17β-estradiol (E2 group; N = 5 eels), and co-implanted 17 MT and E2 (17 MT + E2 group; *N* = 5 eels). Observations are sorted from lowest to highest observation for each group within each measure.

Histological slides of ovarian tissue showed that oocytes from eels increased in diameter during the simulated migratory period, from 107 ± 3 μm before, to 122 ± 4 μm after swimming. At the end of the implantation period, diameters in the control group were the smallest (157 ± 5 μm), larger diameters were observed in groups 17 MT (211 ± 6 μm) and E2 (226 ± 7 μm), and largest measures were made in the 17 MT + E2 group (277 ± 11 μm) (Figure 5). Statistical analyses indicated that diameters differed significantly when compared across all groups (*p* < 0.001), as well as when pre- and post-migration were excluded (*p* < 0.001; Figure 6). Small yolk granules were observed in oocytes from eels of the E2 and 17 MT + E2 groups, but not for eels of the 17 MT-group and the controls. The centred lipid globules were larger in the steroid-treated eels of the 17MT, E2, and 17 MT + E2 groups in comparison with controls. Mean lipid diameter was significantly larger in the steroid-implant treated eels (17 MT: 14.54 ± 0.30 μm; E2: 12.59 ± 0.26 μm; 17 MT + E2: 12.66 ± 0.38 μm vs. C: 10.64 ± 0.46 μm) than in the pre-migration or post-migration groups of eels (pre-migration: 7.73 ± 0.19 μm; post-migration: 7.84 ± 0.38 μm)(*p* < 0.001; Figure 6). There was a near-significant main effect of implant type on lipid droplet diameter (*p* = 0.07; Figure 6). As lipid diameter correlated positively to oocyte diameter (r = 0.812; *p* < 0.001), and implant type significantly affected oocyte diameter, an effect of implant type on lipid droplet diameter is expected.

### 3.6 Gene expression

**FIGURE 7:**
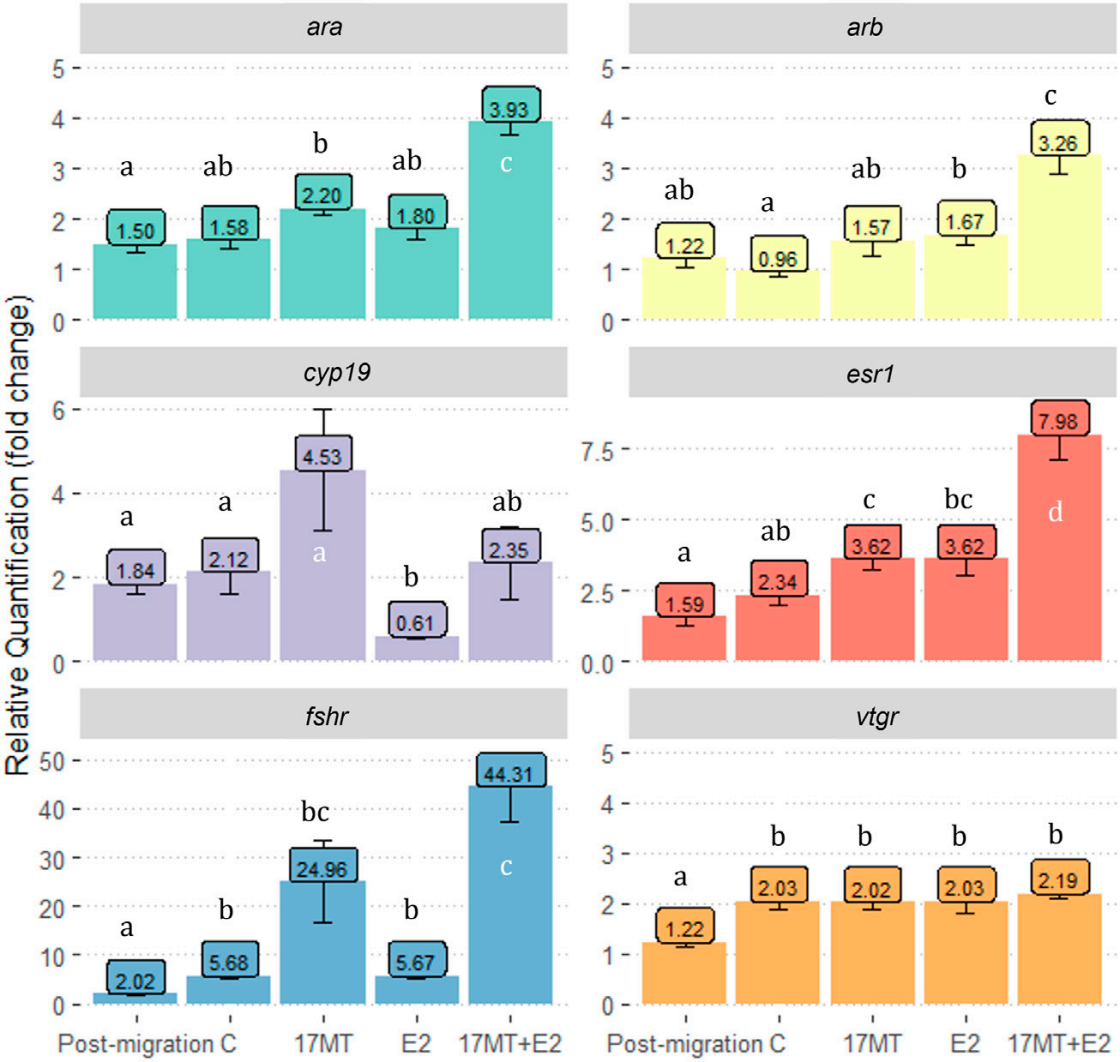
Gene expression of gonad androgen receptor-α (*ara*); androgen receptor-β (*arb*); P450 aromatase (c*yp19*); estrogen receptor 1(*esr1*); follicle-stimulating hormone receptor (*fshr*) and vitellogenin receptor (*vtgr*). Gene expression is shown as relative quantification in “fold change” as compared to gene expression in the pre-migration group. Pre- (*N* = 5) and post-migration (*N* = 5); control (C group; *N* = 5); 17α-methyltestosterone (17 MT group; *N* = 5); 17β-estradiol (E2 group; *N* = 5), and co-implanted 17 MT and E2 (17 MT + E2 group; *N* = 5).

For all six genes investigated in this study, significant differences in relative expression between treatment groups were observed when compared with the pre-migration group (*p* < 0.05; Figure 7). Expression of *ara* was significantly higher in the 17 MT + E2 group when compared to other groups (*p* = 0.002). For *arb* (*p* = 0.007) and *esr1* (*p* = 0.0003), similar differences were observed, with the 17 MT + E2 group showing significantly higher expression than in all the other groups. For *cyp19*, the 17 MT group showed the highest expression, although the difference was only significantly when compared with the E2 group (*p* = 0.041) that showed the lowest expression (Figure 4). Higher *fshr* expression was observed for 17 and 17 MT + E2 which were not significantly different from each other, but that differed between the 17 MT + E2 and the other implant groups (*p* < 0.001; Figure 7). Expressions of the *fshr* and *vtgr* genes in the pre- and post-migration group were significantly lower than in the controls (Figure 7).

### 3.7 Carp pituitary extract treatment

Eels of the C-group fully matured after 14.6 ± 1.7 weeks. Eels of the 17 MT (*p* = 0.154) and E2-groups (*p* = 0.019) matured faster, requiring 12.4 ± 1.7 and 12.1 ± 1.7 weeks, respectively (Table 3). Finally, eels of the 17 MT + E2 group matured fastest (*p* < 0.001 vs. controls), requiring only 10.4 ± 2.4 weeks. In this experiment, eels treated with 17MT, both in the 17 and 17 MT + E2 groups, showed higher levels of mortality and a lower percentage of eels reached full maturity (Table 3). Of the eels of the 17 and 17 MT + E2 groups, only 31% and 56%, respectively, reached maturity. Of the E2-group, 69% of the eels matured which is comparable to the 75% of the controls.

**TABLE 3:** Effects of carp pituitary extract treatment (CPE) on eels from the different treatment groups with C = control with sham implant; 17 MT = 17α methyltestosterone implant; E2 = 17β-estradiol implant, and 17 MT + E2 = the co-implantation. % matured indicates the percentage of eels that reached full sexual maturation (hydration response and oocyte maturation as indicated by a body weight index of >110) and the number of weeks with weekly CPE injections that the eels needed to reach this stage. Statistical differences between groups which are indicated by letters a-b.

Implant group	Matured (%)	Weeks
C	75^a^	14.6 ± 1.7^a^
17 MT	31^b^	12.4 ± 1.7^ab^
E2	69^ab^	12.1 ± 1.7^b^
17 MT + E2	56^ab^	10.4 ± 2.4^b^

## 4 Discussion

The aim of this study was to assess the separate and combined effects of 17MT, as potent activator of the androgen receptor, and E2, as hepatic vitellogenin inducer, on vitellogenesis during the sexual maturation of feminised European eels. The eels were subjected to a 3,500 km simulated migration followed by a 2-months steroid-implant treatment with 17MT, E2 or a combination of both sex steroids *versus* a sham-implanted group as negative control. Here we discuss the effects of simulated migration and steroid implants on the progression of sexual maturation in European eel. Specifically, the effects of 17 MT on previtellogenesis, the effects of E2 on vitellogenesis, and the synergistic effects were assessed as well as the consequences for weekly CPE injections.

### 4.1 Simulated migration and starvation

After simulated migration, experimental eels showed subtle increases in eye size, GSI and oocyte diameter and a lower HSI in comparison with eels of the pre-migration group. Plasma steroid concentrations also showed marked changes, such as an increase in 11-KT levels and a decrease in E2 levels. The increase in concentration of plasma 11-KT and increase of eye size are conform previous studies that demonstrated that eel silvering is under steroid control (Rohr et al., 2001) and that eye enlargement during silvering is 11-KT mediated (Thomson-Laing et al., 2018). 11-KT plasma concentrations are generally much higher in silver than in yellow eels (Lokman et al., 1998; Sudo et al., 2011; Jéhannet et al., 2019). Eye enlargement correlates with gonadal development in silver eels (Pankhurst, 1982) as shown by GSI values that increase from values below 1 in European yellow eels up to 2.5 in silver eels. The drop in HSI probably indicated a starvation effect that elicited metabolism of lipoprotein complexes for use in the muscles and ovary (Damsteegt et al., 2015).

Before discussing steroid treatment effects, it must be noted that increases in EI, GSI, oocyte diameter, and relative *fshr*- and *vtgr* expression, also occurred between the post-migration and control group. These observations indicate time/starvation effects, occurring independently from those of 17 MT and E2. The *vtgr* results match observations by Jehannet et al. (2019) and Morini et al. (2020), who reported that *vtgr* transcription already occurred in ovaries of pre-pubertal female European eels. Expression does not further increase in the pre-pubertal silver eel ovary, and is not further enhanced in vitellogenic oocytes, but instead, remains stable. This stabilization of *vtgr* expression after previtellogenesis was also observed in other teleosts (cutthroat trout; Mizuta et al., 2013) and is conform the hypothesis that, during vitellogenesis, the internalized vitellogenin receptor is recycled to the surface of the oocyte for re-use as the oocytes grows.

### 4.2 Effects of 17 MT on oogenesis

Among the eels that received implants, only those in the 17 MT-group showed increased expression of *cyp19* and of *fshr*; indeed, *fshr* expression increased up to 44-fold in the 17 MT + E2 group, reinforcing that 17 MT in combination with E2, specifically, is particularly effective in doing so. The increase in relative *fshr* expression in the 17 MT-group suggests increased FSH sensitivity of the ovary and is suggested to be part of the activation of the BPG, early on in the steroidogenic cascade (Reid et al., 2013). Increased *fshr* expression has also been observed in shortfinned eel, in which 11-KT dramatically increased *fshr* transcript abundance during pre- and early vitellogenic growth (Setiawan et al., 2012). FSH, after binding to its ovarian receptor, is known to initiate vitellogenesis, as it controls E2 biosynthesis by stimulating aromatase activity (Nagaharna et al., 1995). Our results indicate that eels that received a 17 MT implant had the highest mean relative expression of *cyp19* (fold change = 4.53) as compared to eels at the pre-migration stage, and to eels subjected to treatment with an E2 implant where *cyp19* expression is down-regulated vs. the control group. However, despite elevated *cyp19* expression, plasma concentrations of E2 remained very low for eels in the 17 MT-group, suggesting these fish were still in a previtellogenic state. This notion was confirmed by histological analysis, which failed to detect yolk granules in oocytes from 17 MT-implanted eels, likely marking the absence of circulating FSH. Instead, implantation with 17 MT resulted in increased oocyte diameters and confirmed lipid droplet accumulation, in keeping with earlier assertions that 11-KT in eels plays a key role in lipid accumulation into the oocyte (Endo et al., 2011; Damsteegt et al., 2015).The mortality that we observed may be related to the use of 17MT, either at the applied dose or 17 MT per se, in feminized eels. The synthetic steroid 17 MT can have toxic effects (Rivero-Wendt et al., 2013, 2020). In another experiment that we conducted (Palstra et al., 2018), 17 MT implantation did not lead to increased mortality and other studies did not specifically report on occurrence of mortality in relation to the use of 17 MT (*A. japonica*—Lin et al., 1991; *A. australis*—Lokman et al., 2015; *A. anguilla*—Mordenti et al., 2018). Still, all these studies were executed with wild eels, not with feminized eels. Either applying a lower dose of 17 MT or using 11-KT instead in feminized eels may avoid increased mortality rates which is subject of our current investigations.

### 4.3 Triggering of vitellogenesis by E2

Specific for eels in the estrogen (co)-implanted groups were vitellogenesis-associated changes such as increased HSI, which is suggestive of increased liver activity and vitellogenin production. The mean GSI of the E2-treated group (3.35 ± 0.23) corresponded to values that are typical of eels that are in early stages of vitellogenesis. Interestingly, in the histological images, peripheral yolk granule accumulation in the oocytes could be observed (Figure 5). Thus, the feminized European eels in this study showed E2-induced yolk deposition in the oocytes in contrast to results of earlier studies in European eels (Olivereau & Olivereau, 1979; Burzawa-Gerard and Dumas-Vidal, 1991) and to findings in the shortfinned eels in the recent study of Thomson-Laing et al. (2019). E2-induced yolk deposition may be associated with effects of early life feminization, supporting the hypothesized sensitization of sexual maturation in Japanese eels (Chai et al., 2010; Ijiri et al., 2011). Further research is required into the epigenetic effects of feminization by long-term dietary E2 treatment.

### 4.4 Co-implantation with 17 MT and E2 elicits synergistic effects

Several variables were significantly increased in both the 17 MT and E2 groups as compared to the controls, but eels in the 17 MT + E2 group showed even further increases, indicating a synergistic effect. Such effects were evident for EI, GSI, and oocyte diameter. GSI increased to over three-fold the values seen in controls (17 MT + E2: 6.52 ± 0.42). Similar synergistic effects were observed for HSI, for which the co-implantation elicited the significantly highest values. Histological images illustrated a dramatic increase in the number of yolk granules, at higher density and more central location than the eels that only received E2. A strong synergistic effect was also observed for oocyte diameter and lipid droplet diameter. Oocyte diameter was almost 1.8-fold greater in ovarian tissue of eels of the 17 MT + E2 group in comparison with the controls. Finally, synergistic effects of co-treatment with 17 MT and E2 were also observed for the expression of *esr1* and *fshr*. Taken together, the significant advances in oocyte development suggest that circulating levels of FSH may have increased to successfully induce Vtg uptake after binding to the Fshr. The synergistic effect of co-implantation with 17 MT and E2 in the feminized European eels of this study is in line with the findings in shortfinned eels (Thomson-Laing et al., 2019).

Also, both ovarian androgen receptors in eels from the co-treated group exhibited increased expression as compared to the controls or the eels that received only 17 MT or E2. The increased androgen receptor expression in the 17 MT + E2 group may be related to androgen-mediated VTG synthesis (Kwon and Mugiya, 1994; Peyon et al., 1996; Asanuma et al., 2003). Considering the positive effects of 17 MT on *esr1* expression, and of E2 on *arb* expression, it may even be that there is a cross-linked stimulation explaining the synergistically stimulating effect of 17 MT and E2 co-implantation on yolk- and lipid accumulation and the advanced oocyte development that we observed. Still, little is known about non-classical steroids and their roles during oogenesis.

### 4.5 Steroid biology during (induced) oogenesis in european eel

We employed LCMS to observe changes in steroid profiles in the blood of feminized European eels associated with simulated migration and steroid treatment. Simulated migration generally reduced the levels of focal steroids in the blood stream, but resulted in a small increase in plasma levels of 11-KT. Our LCMS study resulted in identification of detectable amounts of 5α- and 5β-androstanediols, whereas 5α-DHT was not detected. Quérat et al. (1987) similarly reported on the absence of 5α-DHT and the presence of 5α-androstane-3β,17β-diol, albeit at lower levels (∼ 0.2 ng ml^−1^), but not 5α-androstane-3α,17β-diol, in female European eels in fresh or sea water. These 5α/β-androstanediols are metabolites of testosterone and 5α/β-dihydrotestosterone, two (T, 5α-DHT) of which are potent androgens in the eel (e.g., Todo et al., 1999). Whilst the ability of these diols to transactivate the eel androgen receptor is not, to our knowledge, currently known, it is significant that these metabolites (or at least, 5α-androstane-3α,17β-diol) can be converted by a suite of cell lines into 5α-DHT—in doing so, minimal, if any, AR transactivation (5α-androstane-3α,17β-diol) can be dramatically increased (Zimmerman et al., 2004; Mohler et al., 2011). Aside from (some) AR transactivation, 5α-androstane-3α,17β-diol could bind to mammalian estrogen receptor (human, rat) expressed *in vitro*, albeit at much lower affinity (14–30 fold) than E2 (Kuiper et al., 1997).

The current observations are interesting, as 5α/β-reduced androgens were also previously documented after gas chromatography-MS from wild silver New Zealand eel females (both shortfinned eels, *A. australis*, and longfinned eels, *A. dieffenbachii*) but in these species, the 17-ketosteroids (3α/β- and 5α/β-reduction of androstenedione, rather than testosterone) predominated (Lokman et al., 1998). The reason for the apparent predominance of 17β-hydroxy-androgens in the present study, as opposed to 17-ketosteroids in the study on New Zealand eels is not clear, but could simply reflect the limited number of focal 17-ketosteroids in the current study. Alternatively, captivity-induced gonadal arrest (current study) may have resulted in decreased steroidogenesis and increased 3α/β-, 5α/β-, and 17β-reductase activities, conceivably reflected in higher levels of T and 11-KT in wild New Zealand eels. Further study on the effect of captivity on gonadal steroidogenesis and/or steroidogenic arrest are needed to test these ideas.

## 5 Conclusion

In conclusion, E2 is necessary to start vitellogenesis but 17 MT has specific effects on *cyp19* and *fshr* expression. The combined steroid treatment shows synergistic effects although 17 MT implant treatment, followed in time by E2 treatment, may result in a more natural sequence of sexual maturation. As such, steroid implants could be applied in assisted reproduction protocols for European eel as slow-release systems to initiate vitellogenesis and to reduce the number of weekly CPE injections, potentially improving oocyte quality and leading to production of more viable and robust larvae. Indeed, the combined 17 MT + E2 treatment reduced the number of weeks of weekly PE injections from ∼14 down to ∼10 weeks, similar to a recent study in shortfinned eels (Damsteegt et al., 2020 using salmon PE). Still, because in this study 17 MT was likely associated with increased mortality, a lower dose of 17MT, or 11KT, may prove to be a better option for application.

As for steroid biology, the concentrations of almost all focal steroids decreased with simulated migration and steroid treatment. The presence of a suite of 3α/β-, 5α/β-reduced androgens with low (if any) androgenic activity may be indicative of reduced steroid signaling. This is corroborated by decreases in levels of T, 11-KT, and E2, notwithstanding the notable differences in levels of these steroids when compared to other studies in which they also decreased. Lastly, the use of exogenous steroids, E2 and/or 17MT, resulted in wholesale reductions in levels of steroids in all classes.

## Data Availability

The original contributions presented in the study are included in the article, further inquiries can be directed to the corresponding author.
