# Supplementary material for: Steroid implants for the induction of vitellogenesis in feminized European silver eels (Anguilla anguilla L.)
Source: Front Genet. 2022 Aug 17;13:969202. doi: 10.3389/fgene.2022.969202 (PMC9428156; doi:10.3389/fgene.2022.969202)
Supplement: Supplementary file 1 [file DataSheet1.docx]

Supplementary Material S1

1. $Condition factor \left( K \right)=\left( \mathrm{BW}/\left( \mathrm{BL}^{3} \right) \right)\times100$

$BW: body weight (g), BL: body length (cm)$

1. $Body girth index \left( \mathrm{BGI} \right)={(BG}/{BL) x 100}$

BG: body girth (cm), BL: body length (cm)

1. $Eye index (EI)=100 \times(\left( \left( EDv+EDh \right)\times0.25 \right)^{2}\pi\times\left( 10\times BL \right)$

$EDv: eye diameter vertical \left( \mathrm{mm} \right), EDh:eye diameter horizontal \left( \mathrm{mm} \right)$, $BL:body length (cm)$

1. $Pectoral fin index \left( \mathrm{PFI} \right)=(\mathrm{PFL}/{BL)} x 100$

PFL: pectoral fin length (cm), BL: body length (cm)

1. $Gonadosomatic index (GSI)=(\mathrm{GW}/{BW)} \times100$

$GW: gonad weight (g), BL: body length (cm)$

1. $Hepatosomatic index (HSI)=(\mathrm{LW}/{BW)} \times100$

$LW: liver weight (g), BL: body length (cm)$
